# Androgen receptor expression is associated with a distinct low-proliferative phenotype in triple-negative breast cancer

**DOI:** 10.21203/rs.3.rs-11368679/v1

**Published:** 2026-11-01

**Authors:** Kei KAWASHIMA, Reika Matsushita, Eslam A. Elsaedy, Amanda Copeland, Masanori Oshi, Akimitsu Yamada, Kazutaka Narui, Takashi Ishikawa, Itaru Endo, Kazuaki Takabe

**Affiliations:** Roswell Park Comprehensive Cancer Center; Roswell Park Comprehensive Cancer Center; Roswell Park Comprehensive Cancer Center; Roswell Park Comprehensive Cancer Center; Yokohama City University: Yokohama Shiritsu Daigaku; Yokohama City University: Yokohama Shiritsu Daigaku; Yokohama City University Medical Center: Yokohama Shiritsu Daigaku Fuzoku Shimin Sogo Iryo Center; Tokyo Medical University: Tokyo Ika Daigaku; Yokohama City University: Yokohama Shiritsu Daigaku; Roswell Park Comprehensive Cancer Center

**Keywords:** triple-negative breast cancer, androgen receptor, apocrine, tumor microenvironment, transcriptome

## Abstract

**Background:**

AR-related molecular subtypes, such as the luminal androgen receptor (LAR) subtype, exhibit distinct biological phenotypes in triple-negative breast cancer (TNBC), but the significance of AR expression as a single gene, particularly at single-cell resolution, remains incompletely understood.

**Methods:**

We analyzed four bulk TNBC cohorts (TCGA, n = 170; METABRIC, n = 335; SCAN-B, n = 174; ORIEN, n = 483) and two single-cell RNA-sequencing cohorts (GSE246613, n = 21; Xu et al., n = 26). Bulk analyses evaluated molecular subtypes, proliferation-related pathways, genomic features, immune-cell abundance, and survival. Single-cell analyses assessed epithelial cell states, pathway activity, cell-cycle characteristics, and cell–cell communication.

**Results:**

AR-high tumors were enriched for LAR and molecular apocrine subtypes and had lower histologic grade. AR-expressing epithelial cells showed reduced proliferative activity, with greater G1-phase representation and lower S- and G2M-phase activity. Consistently, AR-high tumors showed lower MKI67 expression and reduced proliferation-related pathway activity. AR-high tumors also had fewer TP53 mutations and more PIK3CA mutations. TGFβ-related activity was increased in AR-expressing epithelial cells, while AR-high tumors showed distinct immune-cell composition, including lower estimated Th1/Th2 abundance and higher M2 macrophage abundance. AR expression was not independently associated with survival.

**Conclusions:**

AR expression identifies a distinct, low-proliferative biological state in TNBC characterized by altered cell-cycle activity, genomic features, enhanced TGFβ-related signaling, and a specific immune-cell composition.

## Introduction

Triple-negative breast cancer (TNBC) accounts for approximately 10–15% of breast cancers and is characterized by aggressive behavior and poor prognosis[1]. Although classified by the absence of estrogen receptor (ER), progesterone receptor (PR), and human epidermal growth factor 2 (HER2), TNBC is biologically heterogeneous[2], and transcriptomic studies have identified several molecular subtypes with distinct biological features within TNBC[3]. Among these, the luminal androgen receptor (LAR) subtype described by Lehmann et al. is defined by an androgen receptor (AR)-related signaling and a luminal-like gene-expression profile[4], distinguishing it from the predominantly basal-like biology of the other TNBC subtypes. AR is a steroid hormone receptor closely associated with apocrine differentiation[5] and is commonly evaluated in breast cancer pathology. Consistent with the distinct biology of AR-related molecular subtypes, AR positivity assessed as a single marker has also been associated with distinct clinicopathological and molecular features in TNBC[6]. Nevertheless, the biological significance and clinical utility of AR in TNBC remain incompletely established.

Immunohistochemistry (IHC) studies have reported AR positivity in approximately 10–40% of TNBCs[7], although the reported prevalence varies depending on the antibody and positivity cutoff used[8]. AR-positive TNBC has been associated with less aggressive proliferative features, including lower ki67 expression[9] and lower histologic grade[10], as well as luminal-like features[11]. Despite these reported biological characteristics, AR status has not been incorporated into routine clinical decision-making for TNBC. Furthermore, although AR signaling is an established therapeutic target in prostate cancer[12], the clinical benefit of AR-targeted therapy in TNBC remains uncertain[13, 14]. Also, most previous studies evaluating AR in TNBC have relied on IHC-based protein assessment, which is less suited to standardized analysis across large-scale data.

With the growing availability of large bulk and single-cell transcriptomic data, AR expression level can now be examined systematically across independent cohorts and at cellular resolution. Although AR-related molecular subtypes such as LAR tumors have been well described, the role of AR expression in TNBC biology remains incompletely understood. In addition, IHC-based AR positivity in TNBC is commonly defined using nuclear staining cutoffs of 1% or 10% of tumor cells[9, 15], despite no standardized cutoff established in the literature. Such threshold-based definitions allow tumors with heterogeneous AR expression across cancer cells to be classified as AR-positive. Importantly, the biological characteristics of AR-expressing tumor cells have not yet been well defined at single-cell resolution.

We hypothesized that AR-expressing tumor cells have distinct biological properties and interactions with the tumor microenvironment (TME), which together contribute to a characteristic phenotype of TNBC. In this study, we integrated multiple bulk transcriptomic and single-cell RNA-sequencing cohorts to comprehensively characterize the biological features associated with AR expression in TNBC, including epithelial cell states, genomic characteristics, signaling activity, and TME.

## Material and Methods

### Data acquisition

Clinical, transcriptomic, and genomic data from four independent TNBC cohorts were used for bulk tumor-level analyses. The Cancer Genome Atlas (TCGA; n = 170)[16], Molecular Taxonomy of Breast Cancer International Consortium (METABRIC; n = 335) [17], and Sweden Cancerome Analysis Network-Breast (SCAN-B; n = 174)[18] cohorts were used for clinicopathological characterization, survival analysis, genomic feature assessment, and transcriptomic profiling. The Oncology Research Information Exchange Network (ORIEN; n = 483) cohort was used primarily for transcriptomic analyses.

Clinical, transcriptomic, and genomic data for TCGA and METABRIC were downloaded from cBioPortal and UCSC Xena. SCAN-B data was obtained from GEO database using the GEOquery package in R. De-identified sequencing data from ORIEN data were obtained through the ORIEN Avatar program under Institutional Review Board-approved protocols at Roswell Park Comprehensive Cancer Center (BDR 198425). For the present study, ORIEN data were used primarily for transcriptomic analyses because several clinicopathological variables were incompletely available.

Single-cell transcriptomic analyses were performed using two publicly available TNBC cohorts: GSE246613 (n = 21)[19] and the single-cell atlas deposited in Zenodo (DOI: 10.5281/zenodo.10672250; n = 26)[20]. GSE246613 was downloaded using the GEOquery package in R, and Zonodo dataset was obtained from the Zenodo repository. All publicly available datasets were de-identified and therefore did not require additional institutional review board approval.

### Molecular subtype classification

For bulk transcriptomic cohorts, molecular subtypes were classified using the PAM50, CIT, and Lehmann TNBC subtype frameworks. PAM50 classification was performed using the genefu package [21] in R, and tumors were classified as Luminal A (LumA), Luminal B (LumB), HER2-enriched (HER2), Basal-like (Basal), or Normal-like (Normal). CIT classification was performed using the citbcmst package[22], and tumors were classified as basal-like (basL), luminal A (lumA), luminal B (lumB), luminal C (lumC), molecular apocrine (mApo), or normal-like (normL). Lehmann TNBC subtypes were classified as basal-like 1 (BL1), basal-like 2 (BL2), immunomodulatory (IM), mesenchymal (M), mesenchymal stem-like (MSL), or luminal androgen receptor (LAR). Associations between AR-high/AR-low groups and molecular subtype distributions were subsequently evaluated.

### Pathway analysis

Differentially expressed genes (DEGs) were identified using the limma package in R. Gene set enrichment analysis (GSEA) was subsequently performed using the fgsea package with the Hallmark gene set collection from MSigDB[23]. Genes were ranked by log_2_ fold-change values, and GSEA was conducted in a pre-ranked manner. Statistical significance was defined as a false discovery rate (FDR) < 0.25, and enrichment magnitude was assessed using the normalized enrichment score (NES). In single-cell analyses, pathway activity was additionally inferred using PROGENy [24] to compare signaling activity between AR-positive and AR-negative epithelial cells.

### Immune and genomic feature analyses

Immune cell infiltration within the TME was estimated using the xCell algorithm. Cytolytic activity (CYT) score was calculated as the geometric mean of granzyme A (GZMA) and perforin (PRF1) expression, as previously described by Rooney et al [25]. Tumor immunogenicity and proliferative activity were assessed using pre-calculated scores reported by Thorsson et al [26].

### Single-cell RNA sequencing analysis

Single-cell RNA sequencing data was processed and analyzed using the Seurat package in R. Cells were normalized, scaled, and clustered using principal component analysis (PCA) and uniform manifold approximation and projection (UMAP). Cell type annotation was performed using the SingleR algorithm. Comparisons between AR-positive and AR-negative epithelial cells were performed using patient-level analyses, with patients containing sufficient numbers of both cell populations included in paired comparisons. Differential expression was assessed using paired patient-by-AR-group pseudobulk profiles with limma-voom, and ranked statistics were used for GSEA with the fgsea package and Hallmark gene sets from MSigDB. PROGENy pathway activity and cell-cycle scores were summarized within each patient before paired comparison between AR groups. Cell-cycle phase and S/G2M scores were calculated using the CellCycleScoring function in Seurat. Cell–cell communication analysis was performed using the CellChat package [27] **separately for individual patients** to infer ligand–receptor interactions and signaling networks between cell populations.

### Statistical analysis

Statistical analyses and data visualization were performed using R (version 4.5.2) with Bioconductor (version 3.21). Group comparisons were performed using the Kruskal-Wallis test, Mann-Whitney U test, or Fisher’s exact test, as appropriate. Survival analyses were conducted using Kaplan-Meier analysis with the log-rank test. A p-value < 0.05 was considered statistically significant for all analyses.

## Results

### Clinicopathological characteristics of TCGA, METABRIC, and SCAN-B cohorts

To characterize the clinicopathological features associated with AR expression in TNBC, clinically determined TNBC patients in TCGA (n = 170), METABRIC (n = 335), and SCAN-B cohort (n = 174) were classified into AR-high and AR-low groups using the cohort-specific median AR expression level. Median age was comparable between the AR-high and AR-low groups across all three cohorts, with no significant difference observed (Table 1). Median age and menopausal status were comparable between the AR-high and AR-low groups across all three cohorts with no significant difference observed (Table 1). Tumor size, similarly, did not significantly differ between the three groups. In contrast, histologic grade was significantly lower in the AR-high group across all three cohorts (TCGA, p = 0.001; METABRIC, p = 0.013; SCAN-B, p = 0.005). Lymph node involvement was more frequent in the AR-high group across all cohorts, however only reached statistical significance in two of the three cohorts (METABRIC, p = 0,026; SCAN-B, p = 0,020).

Notably, AR-high tumors showed markedly distinct molecular subtype distributions. PAM50 differed significantly between AR-high and AR-low tumors across all three cohorts (all p < 0.001). Nearly all AR-low tumors were basal-like across the three cohorts (TCGA, 100%; METABRIC, 95%; SCAN-B, 99%) whereas the proportion of basal-like tumors was substantially lower in the AR-high group (72%, 61%, and 64%, respectively). In the Lehmann classification, the LAR subtype was observed exclusively in the AR-high group, accounting for 25%, 28%, and 32% of AR-high tumors in TCGA, METABRIC, and SCAN-B, respectively (all p < 0.001). A similar pattern was observed in the CIT classification, where the molecular apocrine subtype was markedly enriched in the AR-high group (24%, 45%, and 37%, respectively), whereas the basal-like subtype predominated in the AR-low group (100%, 95%, and 99%, respectively; all p < 0.001). Morphologically, tumors with apocrine features involving ≥ 50% of the tumor were more frequent in the AR-high group than in the AR-low group (9.9% vs. 5.1%), although the overall distribution of apocrine morphology did not differ significantly between the groups (p = 0.70).

Clinical outcomes were compared between the AR-high and AR-low groups across the three cohorts. No significant differences were observed in DFS, DSS, or OS (Supplementary Figure S1). Although OS tended to be more favorable in the AR-high group across cohorts, the differences did not reach statistical significance. Similarly, in multivariable Cox regression analyses adjusted for age, tumor size, lymph node status, histologic grade, and MKI67 expression, AR expression was not independently associated with OS in any cohort.

#### Single-cell analysis reveals distinct signaling activity in AR-positive epithelial cells without major differences in tumor microenvironment composition

To investigate AR expression at the single-cell level, we analyzed two independent single-cell RNA-seq cohorts of treatment naïve TNBCs; GSE246613 (n = 21) and Xu et al. cohort (n = 26; Fig. 1A). In both cohorts, AR expression was predominantly restricted to epithelial cells, fibroblasts, and perivascular-like (PVL) cells, with the highest fraction of AR-positive cells observed in the epithelial cells (Fig. 1B, C). Among epithelial cells subset in GSE246613, no AR-expressing cells were detected in 7 of 21 patients (patients #1–7 in Fig. 1D), whereas the remaining 14 patients (patients #8–21) showed variable proportions of AR-positive epithelial cells, ranging from 1.5% (patient #8) to 66.4% (patient #21). The relative abundance of non-epithelial cell populations showed no clear association with the proportion of AR-expressing epithelial cells across patients (Fig. 1D).

To further characterize the biological differences between AR-positive and AR-negative epithelial cells, pathway activity was inferred using the PROGENy algorithm and compared at the patient level (Fig. 1E). In addition to the expected increase in androgen pathway activity, AR-expressing epithelial cells showed significantly higher inferred TGFβ and p53 pathway activities in both cohorts. In contrast, PI3K, EGFR, and estrogen pathway activities were significantly higher in AR-negative epithelial cells. Together, these findings indicate substantial interpatient heterogeneity in the proportion of AR-expressing epithelial cells in TNBC. While this variation was not clearly associated with the overall composition of non-epithelial cell populations, AR expression was associated with intrinsic differences in epithelial cell biology.

### AR expression is associated with lower proliferative activity in TNBC

Given that AR-high tumors were associated with lower histologic grade and distinct PAM50 molecular subtype, we next examined its proliferative activity. In Hallmark GSEA comparing AR-positive and AR-negative epithelial cells in single cell cohorts, proliferation-related pathways, including E2F targets, G2M checkpoint, MYC targets v1/v2, and mitotic spindle, were predominantly enriched in AR-negative epithelial cells (Fig. 2A). E2F targets, G2M checkpoint, and MYC targets v1 were significantly enriched in AR-negative epithelial cells in both cohorts (FDR < 0.25), whereas MYC targets v2 was enriched only in GSE246613 (FDR = 0.14 vs. 0.95 in Xu et al.). Mitotic spindle showed cohort-specific enrichment. Consistently, cell-cycle analysis using CellCycleScoring function in Seurat package in R showed that AR-positive epithelial cells had a higher mean patient-level proportion of cells in G1 phase than AR-negative epithelial cells in both GSE246613 (79.7% vs. 64.8%) and Xu et al. (80.5% vs. 69.5%), with correspondingly lower proportions of cells in S and G2M phases (Fig. 2B). Patient-level paired comparisons further demonstrated significantly lower G2M and S-phase scores in AR-positive epithelial cells in both GSE246613 (G2M, p = 0.022; S, p = 0.022) and Xu et al. (G2M, p = 0.007; S, p = 0.037; Fig. 2C).

This finding was further validated in large bulk transcriptomic cohorts. Ki67 gene (MKI67) expression, a marker of cellular proliferation, was lower in AR-high tumors across all four cohorts and reached statistical significance in ORIEN, METABRIC, and SCAN-B cohorts (all p < 0.05), but not in TCGA (p = 0.33; Fig. 2D). Similarly, GSEA demonstrated consistent enrichment of proliferation-related pathways in AR-low tumors across four cohorts, with all comparisons meeting the FDR < 0.25 except for mitotic spindle in ORIEN (Fig. 2E).

Overall, these findings indicated that AR-positive epithelial cells demonstrate lower proliferative activity than AR-negative cells. This relationship is replicated at the whole bulk tumor level, with AR-high tumors exhibiting a less proliferative phenotype.

### AR-high TNBC exhibits distinct genomic alterations and reduced genomic instability

Because proliferative activity is closely linked to genomic instability and accumulation of somatic alteration, we next investigated whether AR expression was associated with genomic features in TNBC. In TCGA, AR-high tumors showed significantly lower homologous recombination deficiency (HRD) scores and altered genome fractions than AR-low tumors (both p < 0.05; Fig. 3A). In contrast, non-silent and silent mutation burden, predicted neoantigen load, and intratumoral heterogeneity did not significantly differ between the AR-high and AR-low groups.

For individual mutations, TP53 mutations were more frequent in AR-low tumors, whereas PIK3CA mutations were more frequent in AR-high tumors (p < 0.05; Fig. 3B).

### AR-high TNBC is associated with enhanced TGFβ signaling

Because TGFβ pathway activity was increased in AR-positive epithelial cells (Fig. 1E), we further examined TGFβ-related signaling. Paired patient-level PROGENy analysis demonstrated significantly higher inferred TGFβ pathway activity in AR-positive than in AR-negative epithelial cells in both GSE246613 (p = 0.022) and Xu et al. (p = 0.018; Fig. 4A). CellChat analysis performed at the patient level further suggested preferential TGFβ-mediated communication toward AR-positive cancer cells, with TGFB1-TGFBR1/2 representing the predominant predicted ligand–receptor interaction (Fig. 4B). The strongest inferred TGFB1 signaling originated primarily from vascular and immune cell populations, including endothelial, dendritic, myeloid, and T/NK cells.

TGFβ-related pathways showed positive enrichment in AR-high tumors across all four cohorts, reaching FDR < 0.25 in ORIEN and TCGA (Fig. 4C). Multiple TGFβ ligands, receptors, and downstream response genes showed higher expression in the AR-high group across the cohorts (Fig. 4D). Overall, these results support an association between AR expression and increased TGFβ-related activity at both the epithelial-cell and bulk-tumor levels, with exploratory single-cell communication analysis suggesting a potential contribution of microenvironmental TGFβ signaling.

### AR-high TNBC exhibits an immunosuppressive microenvironment characterized by reduced Th1/Th2 cells and increased M2 macrophages

We next examined the association between AR expression and the immune microenvironment using bulk transcriptomic cohorts. Immune-related Hallmark pathways, including interferon-alpha/gamma response, allograft rejection, and inflammatory response did not show consistent differences or directional trends between AR-high and AR-low tumors across cohorts (Fig. 5A). Similarly, CYT score did not consistently differ significantly between groups (Fig. 5B).

In contrast, xCell-based cell deconvolution analysis revealed more consistent differences in specific immune-cell populations. AR-high tumors showed significantly lower estimated Th1 and Th2 cell infiltration across all cohorts (all p < 0.05; Fig. 5C), whereas M2 macrophage abundance was higher in the AR-high group (all p < 0.05). These findings suggest that AR-high TNBC is associated with a distinct immune-cell composition characterized by reduced estimated Th1/Th2 abundance and increased M2 macrophage abundance.

## Discussion

In this study, we comprehensively characterized the biological features associated with AR expression in TNBC using multiple bulk transcriptomic and single-cell RNA-sequencing cohorts. AR-high tumors were enriched for LAR and molecular apocrine subtypes. AR expression was observed predominantly in tumor epithelial cells, and subsequently AR-positive epithelial cells were associated with lower proliferative activity. Tumors with high AR expression also exhibited enhanced TGFβ signaling and a characteristic immune-cell composition with reduced Th1/Th2 cells and increased M2 macrophages. Based on these findings, AR expression is associated with a distinct biological phenotype involving both intrinsic tumor-cell characteristics and TME.

AR expression and AR-related signaling have long been recognized as hallmarks of apocrine differentiation in breast cancer[28], and an ER-negative/AR-positive phenotype is commonly used to support the diagnosis of breast apocrine carcinoma[29]. In parallel, transcriptomic classifications have identified AR-related subtypes such as the molecular apocrine subtype in the CIT classification[30] and the LAR subtype in the Lehmann classification[4], both of which are known to be associated with apocrine differentiation[28]. Although morphologically defined apocrine carcinoma and these molecular subtypes are not equivalent entities, and have been shown to share AR-related biological features[28]. Additionally, morphologically defined apocrine TNBC and AR-related molecular subtypes have both been associated with low proliferative activity and favorable clinical outcomes[31]. However, when AR is evaluated as a single marker, its associations with tumor proliferation and prognosis in TNBC have been less consistent[13]. The relationship between AR expression itself and proliferative activity in TNBC therefore remains incompletely understood. Several preclinical observations, however, suggest a potential relationship between AR-related biology and cell-cycle regulation[13]. A study using TNBC cell-line models showed that LAR models were particularly sensitive to CDK4/6 inhibition, suggesting distinct cell-cycle regulation in this subtype[32]. In addition, although the effects of AR signaling on proliferation appear to be context dependent, AR agonist treatment has been reported to induce cell-cycle arrest and inhibit growth in breast cancer models, including TNBC cell lines, supporting the possibility that AR signaling itself may influence cell-cycle regulation[33]. In the present study, single cell analysis showed that AR-positive epithelial cells were predominant in the G1 phase and had lower S- and G2M-phase activity than AR-negative cells. Interestingly, AR-high tumors showed fewer TP53 mutations, whereas AR-positive cells showed higher inferred p53 pathway activity. Given the established role of p53 in cell-cycle arrest and cell death[34], these findings are consistent with the reduced cell-cycle activity observed in AR-positive cells, although a direct mechanistic relationship cannot be inferred from the present study.

Another notable finding in the present study was the association between AR expression and increased TGFβ-related activity. This association was consistently observed in the single-cell analyses, while bulk transcriptomic analyses showed positive TGFβ pathway enrichment across cohorts, reaching the predefined FDR threshold in two cohorts. Previous experimental studies have demonstrated a positive feedback relationship between AR and TGFβ signaling in TNBC, in which TGFβ enhanced AR expression and the AR–TGFβ axis promoted tumor-cell survival under anchorage-independent conditions[35]. In cancer, TGFβ has broad and context-dependent effects on tumor cells and the tumor microenvironment, including suppression of antitumor immune responses[36]. Consistent with this role, AR-high tumors in our study showed lower estimated Th1 and Th2 cell abundance and higher M2 macrophage abundance across multiple cohorts. These immune features are compatible with the enhanced TGFβ signaling observed in AR-high tumors, although their mechanistic relationship remains unclear. TGFβ is also known to exert context-dependent effects during tumor progression, acting as a growth suppressor through cell-cycle arrest and apoptosis in early tumorigenesis, while promoting EMT, invasion, and metastasis at more advanced stages[37]. Given that our cohorts largely consisted of treatment-naïve primary breast cancers, the coexistence of enhanced TGFβ signaling and reduced proliferative activity in AR-high tumors may reflect the context-dependent nature of TGFβ signaling rather than necessarily indicating a more aggressive tumor state.

A strength of this study is that it extends previous observations of AR-related biology by characterizing the biological features associated with AR expression as a single gene and further resolving these features at the single-cell level, while recognizing the close association between AR expression and established AR-related molecular subtypes. This approach identified a low-proliferative phenotype associated with AR expression at both the tumor and single-cell levels. Nevertheless, this study has several limitations. First, this was an observational transcriptomic study, and therefore the biological mechanisms underlying these associations could not be fully elucidated. In addition, pathway activity, cell-cell communication, and immune-cell abundance were computationally inferred from transcriptomic data and require further functional validation. Second, AR expressions were assessed at the mRNA expression level, and its concordance with IHC-based AR protein expression was not directly evaluated. Also, AR-high and AR-low tumors were defined using cohort-specific median expression levels, which represent analytical rather than clinically established cutoffs. Therefore, our findings may not be directly applicable to IHC-based AR positivity in clinical practice. Finally, single-cell RNA sequencing is subject to transcript dropout, and some cells classified as AR-negative may therefore have had low levels of AR expression below the detection threshold.

In conclusion, AR expression was associated with a distinct, low-proliferative phenotype characterized by AR-related molecular subtypes, altered cell-cycle activity, enhanced TGFβ signaling, and a specific immune-cell composition. These findings suggest that AR may help identify a biologically distinct subset of TNBC and may have potential utility in treatment stratification and clinical decision-making.

## Figures and Tables

**Figure 1:**
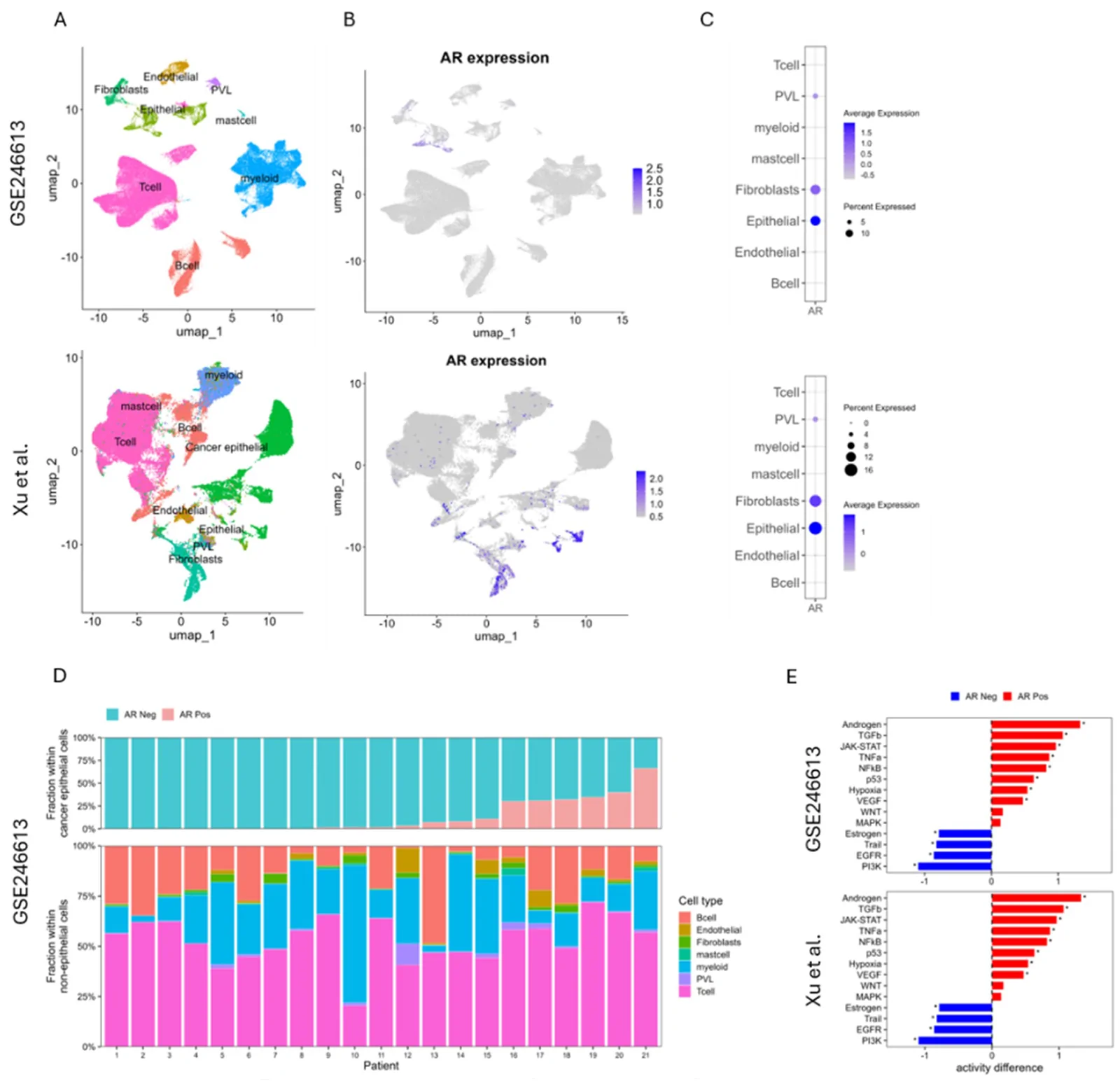
Single-cell characterization of AR expression and associated signaling activity in TNBC. (A) UMAP visualization of major cell populations in two independent TNBC single-cell RNA sequencing cohorts, GSE246613 and the breast cancer single-cell atlas from Xu et al. (B) Feature plots showing AR expression across cell populations in each cohort. (C) Dot plots showing AR expression across annotated cell types. Dot size represents the percentage of AR-positive cells, and color indicates average AR expression. (D) Stacked bar plots showing, for each patient in GSE246613, the proportion of AR-positive and AR-negative epithelial cells (top) and the relative composition of non-epithelial cell populations (bottom). (E) Bar plots showing patient-level differences in inferred pathway activity between AR-positive and AR-negative epithelial cells in the two single-cell cohorts, calculated using PROGENy. Pathway activity was summarized within each patient and compared between AR-positive and AR-negative epithelial cells. Positive values indicate higher pathway activity in AR-positive epithelial cells, whereas negative values indicate higher activity in AR-negative epithelial cells. Statistical significance is indicated by asterisks (*p < 0.05, **p < 0.01, ***p < 0.001).

**Figure 2:**
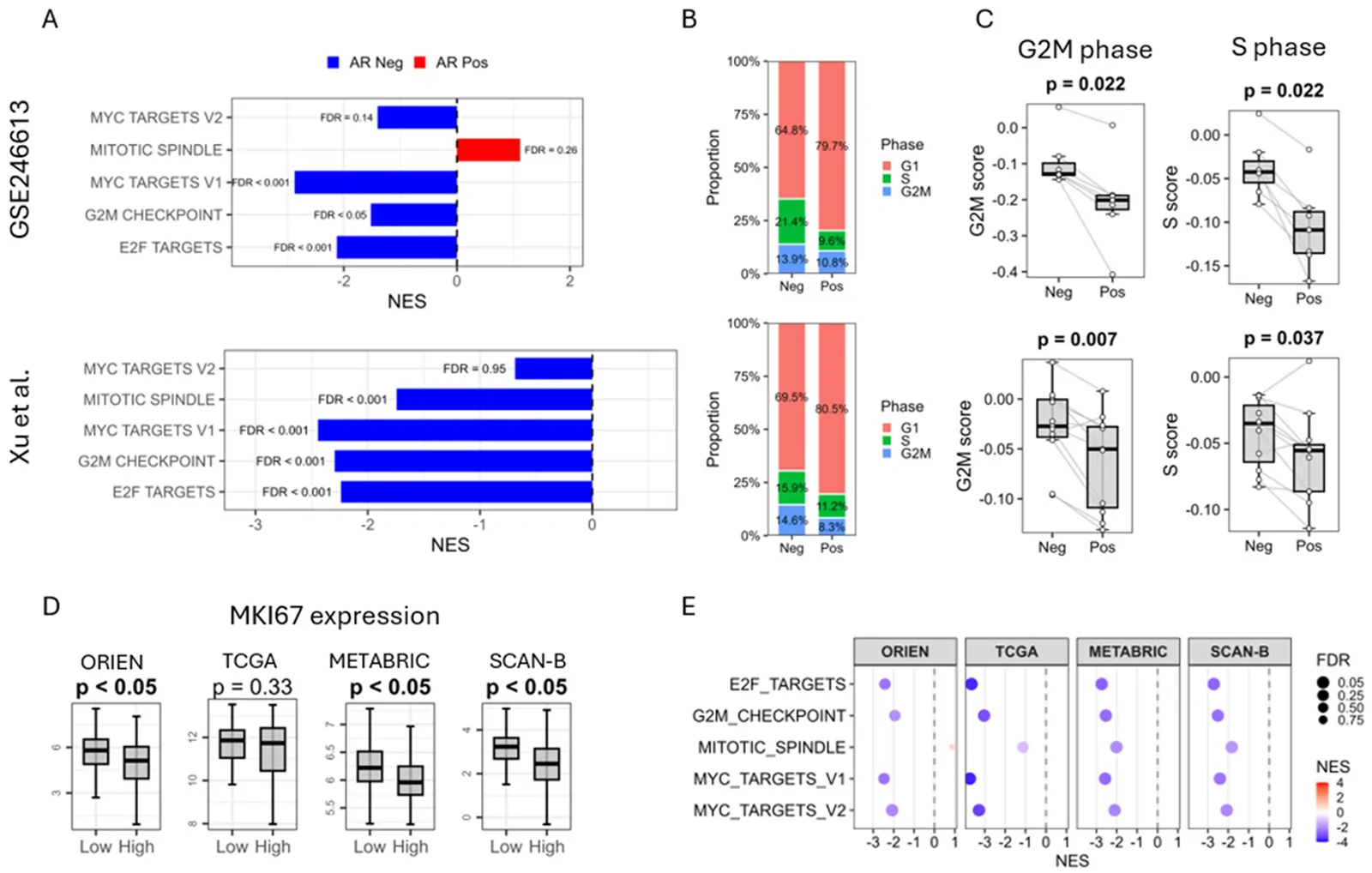
Association of AR expression with proliferative activity in TNBC. (A) Bar plots showing GSEA results for proliferation-related Hallmark pathways comparing AR-positive and AR-negative epithelial cells in GSE246613 and the Xu et al. single-cell cohort, based on paired pseudobulk analysis. Positive normalized enrichment score (NES) values indicate enrichment in AR-positive epithelial cells, whereas negative values indicate enrichment in AR-negative epithelial cells. False discovery rate (FDR) values are shown for each pathway. (B) Stacked bar plots showing the mean proportions across patients of epithelial cells assigned to G1, S, and G2M phases according to AR expression status in the two single-cell cohorts. (C) Paired comparisons of mean G2M and S-phase scores between AR-negative and AR-positive epithelial cells. Each pair of connected points represents one patient. (D) Box plots showing *MKI67* expression according to AR-high and AR-low status in the ORIEN, TCGA, METABRIC, and SCAN-B cohorts. P values calculated using the Wilcoxon test. (E) Dot plots showing GSEA results for proliferation-related Hallmark pathways in AR-high versus AR-low TNBC across the four bulk transcriptomic cohorts. Dot size represents the FDR, and color indicates the NES. Negative NES values indicate enrichment in AR-low tumors.

**Figure 3:**
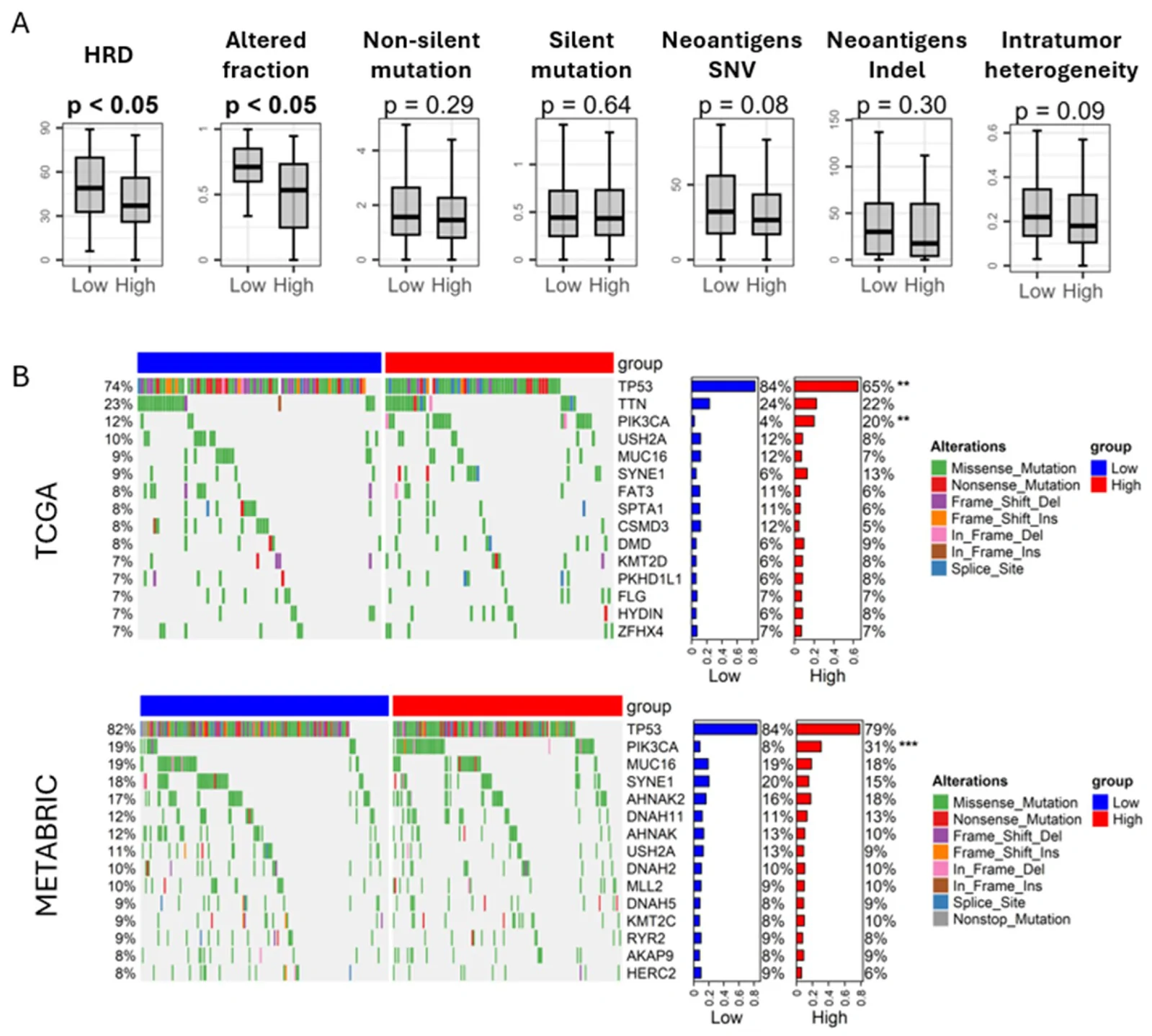
Association of AR expression with genomic features in TNBC. (A) Box plots showing homologous recombination deficiency (HRD) score, altered genome fraction, non-silent mutation burden, silent mutation burden, predicted neoantigen load derived from single nucleotide variants (SNVs) and insertion/deletion mutations (indels), and intratumoral heterogeneity according to AR-high and AR-low status in the TCGA cohort. P values calculated using the Wilcoxon test. (B) Oncoplots showing the most frequently mutated genes in AR-low and AR-high TNBC from the TCGA and METABRIC cohorts. Mutation frequencies shown adjacent to each panel, and mutation types indicated by color. Statistical significance of differences in mutation frequency between AR-low and AR-high tumors was assessed using Fisher’s exact test (*p < 0.05, **p < 0.01, ***p < 0.001).

**Figure 4:**
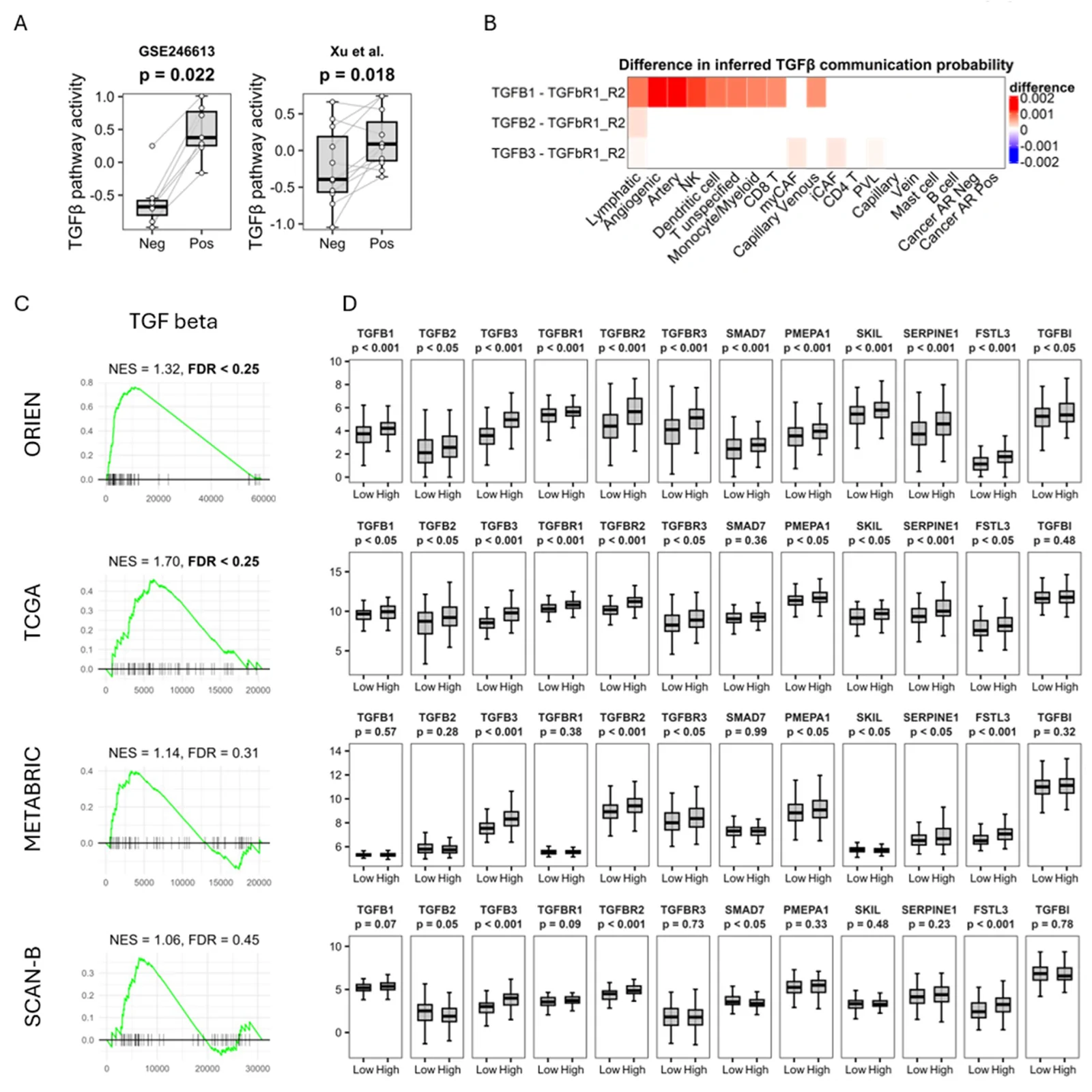
Association of AR expression with TGFβ signaling in TNBC. (A) Paired comparisons of PROGENy-inferred TGFβ pathway activity between AR-negative and AR-positive epithelial cells in GSE246613 and the Xu et al. cohort. Each pair of connected points represents one patient. (B) Heatmap showing differences in inferred TGFβ-mediated cell–cell communication probabilities between AR-positive and AR-negative cancer epithelial cells, calculated using CellChat across individual patients. Positive values indicate higher inferred communication probabilities in AR-positive cancer cells relative to AR-negative cancer cells, whereas negative values indicate the opposite. (C) GSEA enrichment plots showing TGFβ-related pathway enrichment in AR-high versus AR-low TNBC across the bulk transcriptomic cohorts. Normalized enrichment scores (NES) and false discovery rates (FDR) shown for each cohort. (D) Box plots showing the expression of TGFβ ligands, receptors, and downstream response genes according to AR-high and AR-low status across the bulk transcriptomic cohorts. P values calculated using the Wilcoxon test.

**Figure 5:**
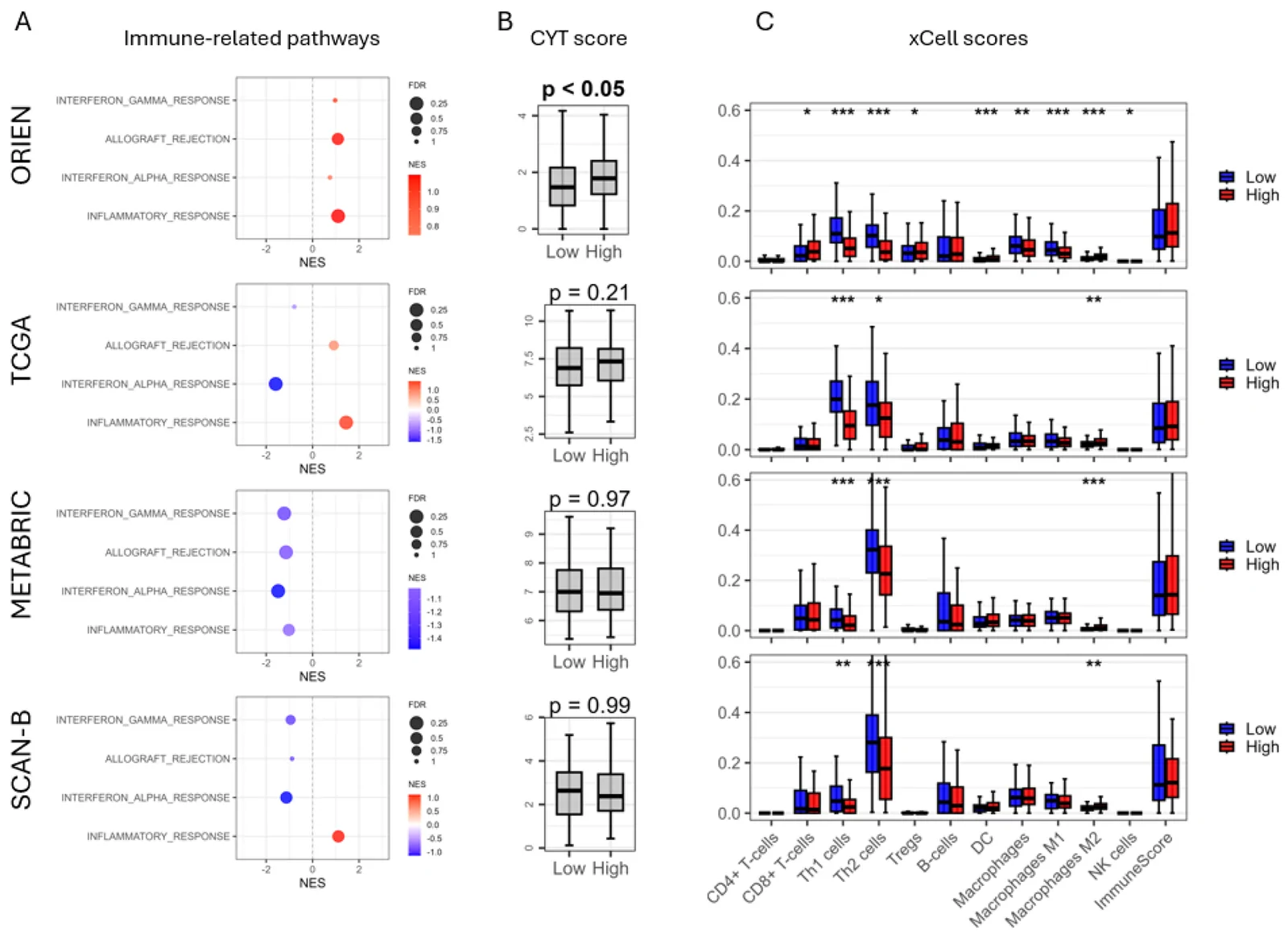
Association of AR expression with immune-related features in TNBC. (A) Dot plots showing GSEA results for immune-related Hallmark pathways in AR-high versus AR-low TNBC across the ORIEN, TCGA, METABRIC, and SCAN-B cohorts. Dot size represents the false discovery rate (FDR), and color indicates the normalized enrichment score (NES). Positive NES values indicate enrichment in AR-high tumors, whereas negative values indicate enrichment in AR-low tumors. (B) Box plots showing cytolytic activity (CYT) scores according to AR-high and AR-low status across the four bulk transcriptomic cohorts. P values calculated using the Wilcoxon test. (C) Box plots showing xCell-estimated immune-cell fractions according to AR-high and AR-low status across the four cohorts. Statistical significance assessed using the Wilcoxon test (*p < 0.05, **p < 0.01, ***p < 0.001).

**Table 1:** Clinicopathological characteristics according to AR expression in TNBC. Clinicopathological characteristics, molecular subtypes, and apocrine morphology were compared between the AR-high and AR-low groups in the TCGA, METABRIC, and SCAN-B cohorts. Continuous variables are presented as median [range], and categorical variables as n (%). P values calculated using the Wilcoxon rank-sum test for continuous variables and Pearson’s chi-squared test or Fisher’s exact test for categorical variables, as appropriate.

Variable	TCGA				METABRIC				SCAN-B			
	all n = 170^1^	Low n = 85^1^	High n = 85^1^	p-value^2^	all n = 335^1^	Low n = 167^1^	High n = 168^1^	p-value^2^	all n = 174^1^	Low n = 87^1^	High n = 87^1^	p-value^2^
Age	55 [29–90]	52 [29–84]	57 [31–90]	0.094	55 [27–96]	55 [28–85]	56 [27–96]	0.12	60 [26–91]	56 [26–91]	63 [27–91]	0.2
Menopause				0.6				0.2				
Post	112 (70%)	55 (72%)	57 (69%)		213 (64%)	100 (60%)	113 (67%)					
Pre	47 (30%)	21 (28%)	26 (31%)		122 (36%)	67 (40%)	55 (33%)					
Grade				< 0.001				0.013				0.005
1	6 (3.8%)	1 (1.3%)	5 (6.2%)		4 (1.2%)	2 (1.2%)	2 (1.2%)		2 (1.1%)	1 (1.1%)	1 (1.1%)	
2	27 (17%)	6 (7.9%)	21 (26%)		43 (13%)	13 (8.1%)	30 (19%)		24 (14%)	5 (5.7%)	19 (22%)	
3	124 (79%)	69 (91%)	55 (68%)		276 (85%)	146 (91%)	130 (80%)		145 (83%)	79 (91%)	66 (76%)	
pT				0.8				0.4				0.4
T1	40 (24%)	20 (24%)	20 (24%)		97 (30%)	43 (26%)	54 (33%)		71 (41%)	32 (37%)	39 (45%)	
T2	110 (65%)	56 (66%)	54 (64%)		199 (61%)	106 (65%)	93 (57%)		83 (48%)	43 (49%)	40 (46%)	
T3	15 (8.8%)	6 (7.1%)	9 (11%)		30 (9.2%)	15 (9.1%)	15 (9.3%)		20 (11%)	12 (14%)	8 (9.2%)	
T4	5 (2.9%)	3 (3.5%)	2 (2.4%)									
pN				0.8				0.026				0.02
N0	111 (65%)	55 (65%)	56 (66%)		149 (48%)	80 (50%)	69 (45%)		106 (63%)	60 (73%)	46 (54%)	
N1	34 (20%)	19 (22%)	15 (18%)		110 (35%)	62 (39%)	48 (31%)		51 (31%)	20 (24%)	31 (36%)	
N2	17 (10%)	8 (9.4%)	9 (11%)		32 (10%)	9 (5.6%)	23 (15%)		10 (6.0%)	2 (2.4%)	8 (9.4%)	
N3	8 (4.7%)	3 (3.5%)	5 (5.9%)		22 (7.0%)	9 (5.6%)	13 (8.5%)					
PAM50				< 0.001				< 0.001				< 0.001
LumA	4 (2.4%)	0 (0%)	4 (4.7%)		0 (0%)	0 (0%)	0 (0%)		1 (0.6%)	0 (0%)	1 (1.1%)	
LumB	1 (0.6%)	0 (0%)	1 (1.2%)		1 (0.3%)	0 (0%)	1 (0.6%)		1 (0.6%)	0 (0%)	1 (1.1%)	
Her2	9 (5.3%)	0 (0%)	9 (11%)		33 (9.9%)	2 (1.2%)	31 (18%)		18 (10%)	0 (0%)	18 (21%)	
Basal	146 (86%)	85 (100%)	61 (72%)		262 (78%)	159 (95%)	103 (61%)		142 (82%)	86 (99%)	56 (64%)	
Normal	10 (5.9%)	0 (0%)	10 (12%)		39 (12%)	6 (3.6%)	33 (20%)		12 (6.9%)	1 (1.1%)	11 (13%)	
Lehmann				< 0.001				< 0.001				< 0.001
BL1	26 (20%)	22 (30%)	4 (7.1%)		74 (26%)	56 (37%)	18 (13%)		28 (20%)	25 (32%)	3 (4.8%)	
BL2	15 (12%)	10 (14%)	5 (8.9%)		21 (7.2%)	10 (6.6%)	11 (7.9%)		9 (6.4%)	2 (2.6%)	7 (11%)	
IM	28 (22%)	19 (26%)	9 (16%)		68 (23%)	41 (27%)	27 (19%)		41 (29%)	23 (29%)	18 (29%)	
M	35 (27%)	21 (29%)	14 (25%)		48 (17%)	35 (23%)	13 (9.4%)		33 (23%)	25 (32%)	8 (13%)	
MSL	11 (8.5%)	1 (1.4%)	10 (18%)		40 (14%)	9 (6.0%)	31 (22%)		10 (7.1%)	3 (3.8%)	7 (11%)	
LAR	14 (11%)	0 (0%)	14 (25%)		39 (13%)	0 (0%)	39 (28%)		20 (14%)	0 (0%)	20 (32%)	
CIT				< 0.001				< 0.001				< 0.001
basL	138 (81%)	85 (100%)	53 (62%)		223 (67%)	158 (95%)	65 (39%)		127 (73%)	86 (99%)	41 (47%)	
lumA	0 (0%)	0 (0%)	0 (0%)		0 (0%)	0 (0%)	0 (0%)		2 (1.1%)	0 (0%)	2 (2.3%)	
lumB	2 (1.2%)	0 (0%)	2 (2.4%)		0 (0%)	0 (0%)	0 (0%)		3 (1.7%)	0 (0%)	3 (3.4%)	
lumC	1 (0.6%)	0 (0%)	1 (1.2%)		22 (6.6%)	2 (1.2%)	20 (12%)		3 (1.7%)	0 (0%)	3 (3.4%)	
mApo	20 (12%)	0 (0%)	20 (24%)		81 (24%)	6 (3.6%)	75 (45%)		33 (19%)	1 (1.1%)	32 (37%)	
normL	9 (5.3%)	0 (0%)	9 (11%)		9 (2.7%)	1 (0.6%)	8 (4.8%)		6 (3.4%)	0 (0%)	6 (6.9%)	
Apocrine morphology				0.7								
Absent	132 (83%)	67 (86%)	65 (80%)									
Minimum	5 (3.1%)	2 (2.6%)	3 (3.7%)									
Moderate	10 (6.3%)	5 (6.4%)	5 (6.2%)									
Marked	12 (7.5%)	4 (5.1%)	8 (9.9%)									

Abbreviations: AR, androgen receptor; TNBC, triple-negative breast cancer; TCGA, The Cancer Genome Atlas; PAM50, Prediction Analysis of Microarray 50; LAR, luminal androgen receptor; BL1, basal-like 1; BL2, basal-like 2; IM, immunomodulatory; M, mesenchymal; MSL, mesenchymal stem-like; CIT, Cartes d’Identité des Tumeurs; basL, basal-like; mApo, molecular apocrine; pT, pathological tumor stage; pN, pathological nodal stage.

1 Median [Min-Max]; n (%)

2 Wilcoxon rank sum test; Pearson’s Chi-squared test; Fisher’s exact test; NA

## Data Availability

Data from TCGA, METABRIC, SCAN-B, GSE246613, and the breast cancer single-cell atlas used in this study are publicly available. TCGA and METABRIC data were obtained from cBioPortal and the UCSC Xena platform. SCAN-B and GSE246613 were accessed through the Gene Expression Omnibus (GEO). The breast cancer single-cell atlas (DOI: 10.5281/zenodo.10672250) was obtained from the Zenodo repository. De-identified clinical, pathological, and sequencing data from the Oncology Research Information Exchange Network (ORIEN) were obtained through the ORIEN Avatar program under Institutional Review Board-approved protocols at Roswell Park Comprehensive Cancer Center and are not publicly available. The code used for the analyses in this study is available from the corresponding author upon reasonable request.
